# Information about changes in platform economy taxation diminishes optimism regarding future use

**DOI:** 10.1007/s40881-024-00160-y

**Published:** 2024-03-14

**Authors:** Jantsje M. Mol, Catherine Molho

**Affiliations:** 1 Center for Research in Experimental Economics and Political Decision Making (CREED), University of Amsterdam, Roetersstraat 11, Postbus 15867, 1001 NJ Amsterdam, The Netherlands; 2 Tinbergen Institute, Amsterdam, The Netherlands; 3 VU Amsterdam, Amsterdam, The Netherlands

**Keywords:** Platform economy, Taxation, Online experiment, Enforced compliance, Voluntary compliance, Trust, C81, D91, H26

## Abstract

Some economic interactions are based on trust, others on monetary incentives or monitoring. In the tax compliance context, the monitoring approach creates compliance based on audits and fines (enforced compliance), in contrast to the trust-based (voluntary compliance) approach, which is based on taxpayers’ willingness to comply. Here, we examine how changes in taxation regarding platform economy revenues affect intended labor supply on such platforms. New EU legislation, effective from 2023, will mandate data sharing between platforms and tax authorities across Europe, thus resulting in increased monitoring. We investigate how this upcoming shift in monitoring power affects the intended use of platforms and how it may interact with users’ trust. We use a survey among platform workers (*N* = 626) in the Netherlands to examine views of the proposed regulation change, corrected for the proportion of platform income and several measures of trust. We experimentally manipulate information by either informing participants about the upcoming monitoring change or not. Results show that informing respondents about the change negatively affects expected supply of labor, and this effect is independent of respondents’ trust. We discuss the policy implications of these results.

**Supplementary Information:**

The online version contains supplementary material available at 10.1007/s40881-024-00160-y.

Some economic interactions are based on trust, others on monetary incentives or monitoring. In the tax compliance context, the traditional monitoring approach creates compliance based on audits and fines, which is known as enforced compliance (Kirchler et al., 2008). While the monitoring and deterrence approach is effective at increasing tax compliance (Alm, 2019; Slemrod, 2019), it may induce reactance and crowd out intrinsic motivation to comply with tax regulations in high-trust contexts (Batrancea et al., 2019; Gangl et al., 2014) and it can be rather costly (Gribnau, 2014). Another promising approach is to promote voluntary compliance, which is based on taxpayers’ willingness to comply and mutual understanding between taxpayers and tax authorities (Boer & Gribnau, 2018; Kirchler et al., 2008). According to the slippery slope framework (Kirchler et al., 2008, 2014), authorities’ power to monitor regulations is key to enforced compliance (in which the taxpayer perceives a monitoring approach by the tax administration), and trust in authorities is key to promote voluntary compliance (in which the taxpayer perceives a service-based approach). Importantly, there may be dynamic effects of power on trust and vice-versa, such that increases in monitoring power may be perceived as an indication that authorities distrust citizens or, to the contrary, as an indication that authorities effectively improve services to taxpayers (Kogler et al., 2013).

In this paper, we examine changes in taxation regarding platform economy revenues. Currently, some European countries have introduced legislation or administrative guidance for platform operators to report information to tax administrations, to prevent unequal competition between the traditional and the platform economy (European Commission, 2020). New EU legislation will be applied from January 2023 onwards, which mandates data sharing between platforms and tax authorities across Europe. As a result, there will be a shift in the taxation approach regarding platform economy revenues, such that the power of tax authorities to monitor earnings will increase. Although such monitoring power may ultimately increase enforced compliance, it can also have unintended effects on intended supply of labor. Specifically, in a country with high levels of trust and *voluntary compliance*, such as the Netherlands, increasing monitoring power could induce reactance and negatively affect platform use (see Batrancea et al., 2019). Alternatively, citizens who already have high trust in authorities might interpret regulation changes in a positive light (Kogler et al., 2013), buffering against negative effects of such changes.

What happens to the intended use of platforms when the monitoring power of tax authorities increases? Here, we examine how providing information (or not) to taxpayers about a shift in monitoring power affects expected supply of labor. Although prior work testing the slippery slope framework has primarily examined tax compliance (Kogler et al., 2013, Kirchler et al., 2014), we instead chose to focus on expected supply of labor as our main outcome of interest for two reasons. Specifically, our decision was informed by the particular characteristics of the upcoming EU regulation change, on the one hand, and platform users, on the other hand. Given that the upcoming regulation change mandates data sharing between platforms and tax authorities, which may be implemented through automatically populating relevant fields in tax declarations, tax authorities’ monitoring power increases to the extent that non-compliance becomes extremely difficult. Combined with the unique characteristics of platform workers, who typically use platforms as a secondary source of income and are more flexible than traditional workers, this implies that negative outcomes of a regulation change would likely manifest in workers’ use of platforms rather than non-compliance with tax authorities. Second, prior work has mainly examined tax compliance in hypothetical scenarios (Batrancea et al., 2019; Kogler et al., 2013), whereas we use a survey experiment with a particular target population. Attempting to directly ask about intended tax compliance among platform workers would not be appropriate in this context, as it would likely result in socially desirable (non-honest) responding.

We further examine whether information about this shift affects intended use of platforms differently depending on whether users’ trust is high or low. Given the link between trust and voluntary compliance (Kogler et al., 2013), we expected that high trust may buffer against the potential negative effects of learning about the EU regulation change. Put differently, we expected that the negative effects of increased tax monitoring on intended future use would be stronger among those platform workers who show low trust, and would, therefore, be less intrinsically motivated to comply with tax regulations in the first place. We consider and measure three types of trust, including generalized trust toward strangers, trust in the Dutch government (i.e., institutions), and trust in digital platforms.

The platform economy (also known as collaborative economy) allows private individuals to temporarily share goods (referred to as sharing economy) or services (referred to as gig economy) through an online marketplace (European Commission, 2016). Existing tax regimes were not designed for transactions provided in the platform economy (Casarico et al., 2017). Currently, many people may not know that they need to pay income tax on their platform revenues. It could also be the case that people are aware of income tax in relation to platform revenues, but that they are not aware of the details, such as the threshold level. Information about tax awareness regarding the platform economy is currently lacking, both regarding taxpayers (providers) as well as users. Tax offices have negotiated at the European Union level with the platforms to get information about revenues made through these platforms. On 22 March 2021, the Council of the European Union adopted new rules revising the Directive on administrative cooperation in the field of taxation (Council Directive 2011/16/EU or DAC) to extend the European Union (EU) tax transparency rules reporting by digital platforms on their sellers (DAC7) (European Union, 2021). This means that all member states should follow the contents of the Directive by creating national legislation and regulations. The Directive states that legislation should start on 1 January 2023, which means that 2023 is the first year in which platforms need to collect and verify data.^1^ They need to send the data before February 2024 to the national tax authorities.

At the same time, the platform economy is on the rise in most European countries (PwC, 2015) as it offers more flexible ways to earn a living than the traditional economy. For example, platform income could be earned by temporary listing your home on Airbnb (sharing economy) or by offering services on Deliveroo and Uber (gig economy). The flexibility offered by the platform economy has clear advantages, i.e., workers are free to choose when and how much to work on a platform, but the clear disadvantage is precarity, in terms of lack of stable employment, benefits, and insurance. This paper assesses the views of gig economy platform users in the Netherlands. According to a recent report of the Dutch Social and Economic Council, almost 1% of the Dutch labor force was active on one of the large gig economy platforms in 2019–2020, and this number is still increasing (ter Weel et al., 2020). The gig economy in the Netherlands is estimated to be between 0.4 and 2.8% of the working population (Rözer et al., 2021).

We administered a survey in collaboration with the Dutch Tax Administration and one of the largest gig economy platforms active in the Netherlands, with approximately 15.000 active users. Within our survey, we include two experimental treatments (Information versus Control), such that we either provide participants with information about the upcoming taxation change or not. Importantly, at the time of data collection and prior to January 2023, little information about this regulation change had been communicated to the general public and platform users (see Appendix D). Therefore, it is highly unlikely that participants in the Control treatment would be aware of the regulation change. The dependent variable of interest is the response to the question of future expected supply (“Do you expect any change in the number of hours you work via platforms in the coming two years?”). Two hypotheses were preregistered before data collection. There are many possible responses to such a change in regulation, both negative (e.g., I need to declare tax, lowering my net platform incomes; I do not like administrative changes) and positive (e.g., it will save some administrative work). We predict that the negative expectations outweigh the positive ones, based on loss aversion (Kahneman & Tversky, 1979). Therefore, our first hypothesis predicts a net negative effect of communicating the regulation change on expected supply^2^:

**H1**: Communicating a regulation change that requires digital platforms to report the income of platform workers to respective national tax authorities negatively affects expected labor supply on these platforms.

One relevant concept in taxation is trust in government (or *authorities*) which affects voluntary compliance as described in the *slippery slope framework* (Kirchler et al., 2008). This could mean that individuals with low trust in government express more negative responses (e.g., because they do not trust that the regulation change will be smooth without administrative hassle) to the communicated regulation change than individuals with high trust in government (e.g., because they trust that the new system will be in their best interest). In contrast, individuals with high trust in government may also express more positive responses to the regulation change, because an increase in the authorities’ monitoring power can act to reassure them that they are protected from potential free riders. Complementarily, individuals with high trust in government may be already intrinsically motivated to comply with tax regulations, which would mute any negative effects of informing them about EU regulations that increase tax authorities’ monitoring abilities. Therefore, we anticipate an interaction effect between the information treatment and self-reported trust in government:

**H2**: The change in expected supply between the Information and the Control treatment is larger for individuals with low trust in government.^3^

## Methodology

We developed a survey in collaboration with the Dutch Tax Administration to examine platform use and responses to regulation change. The survey was administered using Qualtrics software among regular users of the gig economy platform YoungOnes and consisted of several blocks of questions: (a) platform use, (b) trust, (c) knowledge of fiscal regulations, (d) communication about fiscal regulations, (e) expected supply, (f) socio-economic background, and (g) general feedback. Our dependent variable is the expected supply question: “Do you expect any change in the number of hours you work via platforms in the coming two years?” with answers ranging from 1 (*less*) to 2 (*no change*) to 3 (*more*). Each respondent was randomly assigned by the software into one of two treatments: the Control treatment or the Information treatment. In the Information treatment, respondents were informed about the DAC7 regulation change (see Box 1), right before the DV (expected supply) question. Subsequently, these respondents were asked how they thought most other platform workers would respond to this change, in an attempt to minimize demand effects. In this way, respondents with sensitive opinions could hide these in their answer about others. In the Control treatment, the information and expected response of others was simply skipped. The analysis plan was preregistered before the start of the data collection (https://aspredicted.org/6ax87.pdf) and the Faculty’s ethics committee approved the study in January 2022. We measured trust with three distinct survey questions, based on the World Values Survey (Inglehart et al., 2014): general trust (“In general, would you say that most other people can be trusted?”), trust in the government (“Would you say that the Dutch Government can be trusted?”) and trust in platforms (“Would you say that digital platforms can be trusted?”*)*. All trust ratings used response scales from 1 (*strongly disagree*) to 5 (*strongly agree*) allowing for NA (*rather not say).*

*Box 1: Information treatment, translated from Dutch* *In March 2021, the EU passed a law that requires digital platforms to report the income of their platform workers to the respective national tax authorities, starting January 1st, 2023. This information may be used to prepopulate tax forms (starting 2024) similar to the tax forms of citizens in wage labor.”*

The invitation email was sent on Monday February 7th 2022 to approximately 12,000 platform workers who had actively responded to jobs offered (gigs) on the YoungOnes platform in January 2022. A translation of the invitation email can be found in Appendix A. In line with the preregistration, the platform sent a reminder to all workers approximately 1 week later (Wednesday February 16th, 2022). Participation was voluntary and all respondents participated in a lottery for a €20 voucher for an online retail shop. Note that the voluntary survey among actual platform workers did not allow any sample size estimations prior to the experiment. In total, 943 platform workers clicked the link, and 658 respondents completed the survey (response rate 5.48%). In line with the preregistration (following Leiner, 2019 on inattentive respondents), we removed 32 participants who were in the lowest 5% of the time distribution leaving 626 respondents for analysis. The anonymized dataset and full analysis code in R Markdown can be found on OSF (https://osf.io/6w87b). The full transcript of survey questions can be found in Appendix B. A large set of questions is not used in the current analysis, as they were intended to improve the information provision on the YoungOnes / Tax Authority website (e.g., response to the statement “The information I was looking for contained a lot of jargon”).

## Results

Table 1 presents some descriptive statistics of the survey. There are no differences across the treatments on any of these variables. Most platform workers at YoungOnes are young, male students. The sample is very heterogeneous when it comes to the proportion of platform income out of total income.

**Table 1:** Descriptive statistics

	Control (*N* = 313)	Information (*N* = 313)	Total (*N* = 626)
*Gender*			
Female	110 (35%)	99 (32%)	209 (33%)
Male	199 (64%)	208 (66%)	407 (65%)
Other	4 (1%)	6 (2%)	10 (2%)
*Age (years)*			
Mean (SD)	22 (± 6.5)	22 (± 7.2)	22 (± 6.9)
*Main occupation*			
Student	188 (60%)	185 (59%)	373 (60%)
Platform work	39 (12%)	41 (13%)	80 (13%)
Self-employed	32 (10%)	35 (11%)	67 (11%)
Part time	26 (8%)	21 (7%)	47 (8%)
Full time	15 (5%)	14 (4%)	29 (5%)
Other	13 (4%)	17 (5%)	30 (5%)
*Net household income/month*			
Mean (SD)	2500 (± 2000)	2300 (± 1900)	2400 (± 1900)
Rather not say	74 (23.6%)	74 (23.6%)	148 (23.6%)
*Hours/week work via platform*			
Mean (SD)	11 (± 9.3)	12 (± 10)	12 (± 9.9)
*Hours of workweek via platform*			
Zero	29 (9%)	28 (9%)	57 (9%)
Less than half	78 (25%)	75 (24%)	153 (24%)
Half	53 (17%)	56 (18%)	109 (17%)
More than half	59 (19%)	68 (22%)	127 (20%)
Only income	94 (30%)	86 (27%)	180 (29%)
*General trust*			
Mean (SD)	3.6 (± 0.86)	3.6 (± 0.86)	3.6 (± 0.86)
Rather not say	8 (2.6%)	8 (2.6%)	16 (2.6%)
*Trust in government*			
Mean (SD)	3.4 (± 1.0)	3.4 (± 1.0)	3.4 (± 1.0)
Rather not say	11 (3.5%)	12 (3.8%)	23 (3.7%)
*Trust in platforms*			
Mean (SD)	3.4 (± 1.0)	3.4 (± 1.0)	3.4 (± 1.0)
Rather not say	11 (3.5%)	12 (3.8%)	23 (3.7%)
*Self-reported tax knowledge*			
Mean (SD)	3.6 (± 0.82)	3.7 (± 0.89)	3.6 (± 0.86)

Our main hypothesis concerns the effect of information on expected supply. First, we examined how respondents in the information treatment perceived the expected regulation change. When asked *What do you think most gig workers think of this?* (multiple answers possible), the most frequently selected answer (25%) was positive (“this saves administrative work”), followed by 17% who perceived the change as unnecessary, 11% who perceived it as promoting tax equality, and 10% who perceived it as potentially increasing their tax burden. As respondents could select multiple answers, it is interesting to sum the results by direction: 10% selected two positive answers, 42% selected one positive answer, 36% selected one negative answer, and 5% selected two negative answers. Only 7% selected both positive and negative answers. These results do not confirm our original conjecture that the negative arguments would outweigh the positive ones following loss aversion. Instead, the two distinct groups are in line with regulatory focus theory, which states that individuals can have two distinct motivational systems—promotion focus and prevention focus (Higgins, 1997).

**Fig. 1:**
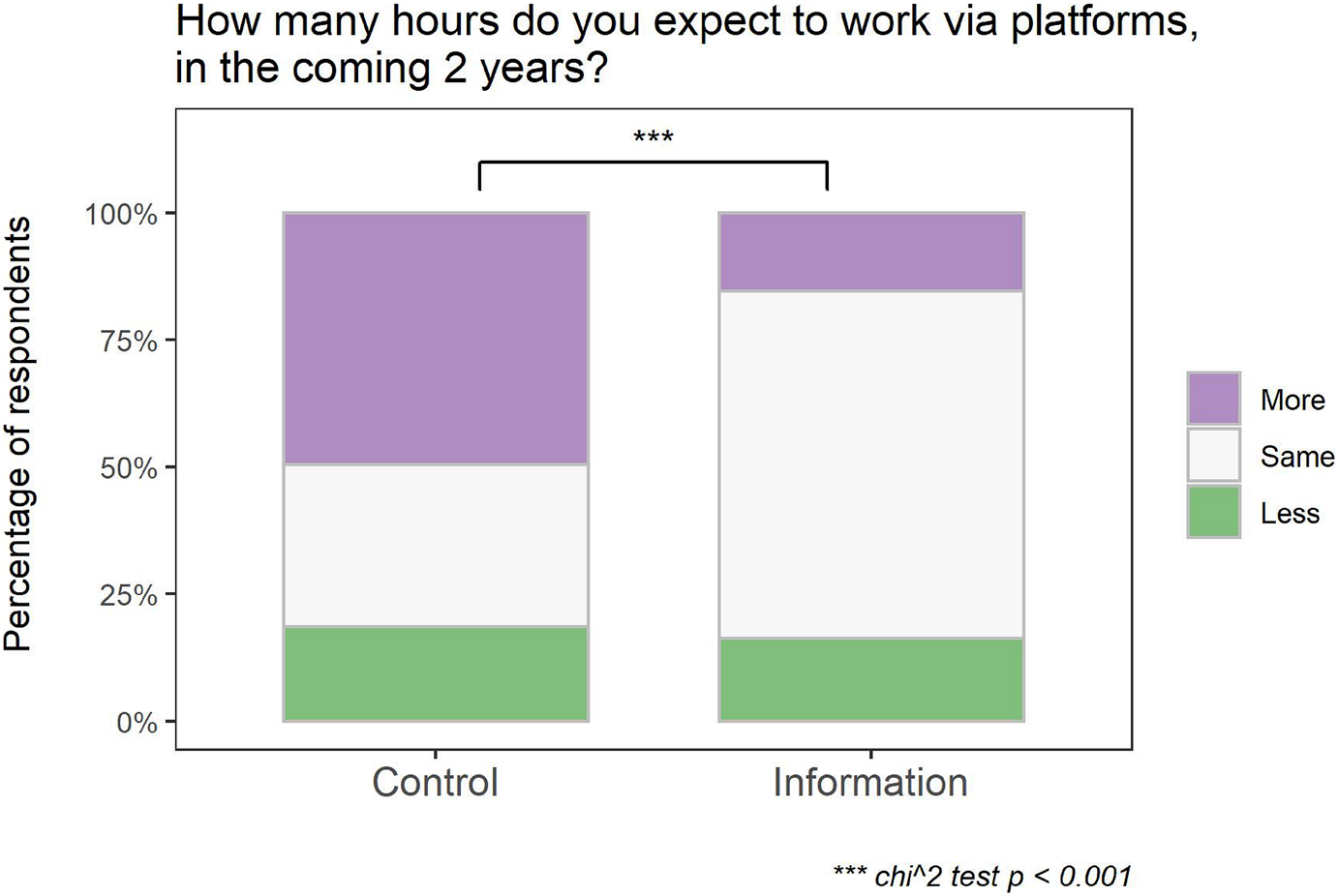
Main treatment effect

Overall, a small group of respondents (17%) indicate they expect to work less via platforms in the coming 2 years, versus a larger group who expects to work more (33%) and half of the respondents (50%) who expect no change at all. Figure 1 shows results separated by treatment. The proportion of respondents who expect to work less is quite similar across treatments, but a large shift occurs between the other two answer categories: in the Control treatment, half of the sample (50%) expects to work more via platforms in the future, compared to only 15% in the Information treatment, where the group who expects to work similar hours is larger (68%) than in the Control treatment (32%). In the exploratory analysis, we examine a possible mechanism behind this effect. First, as preregistered, we examine the change in expected supply across treatments with a χ^2^ test, confirming Hypothesis 1: communicating the regulation change negatively affects expected supply (χ^2^ = 98.237, *de* = *2, p* < 0.001).

**Fig. 2:**
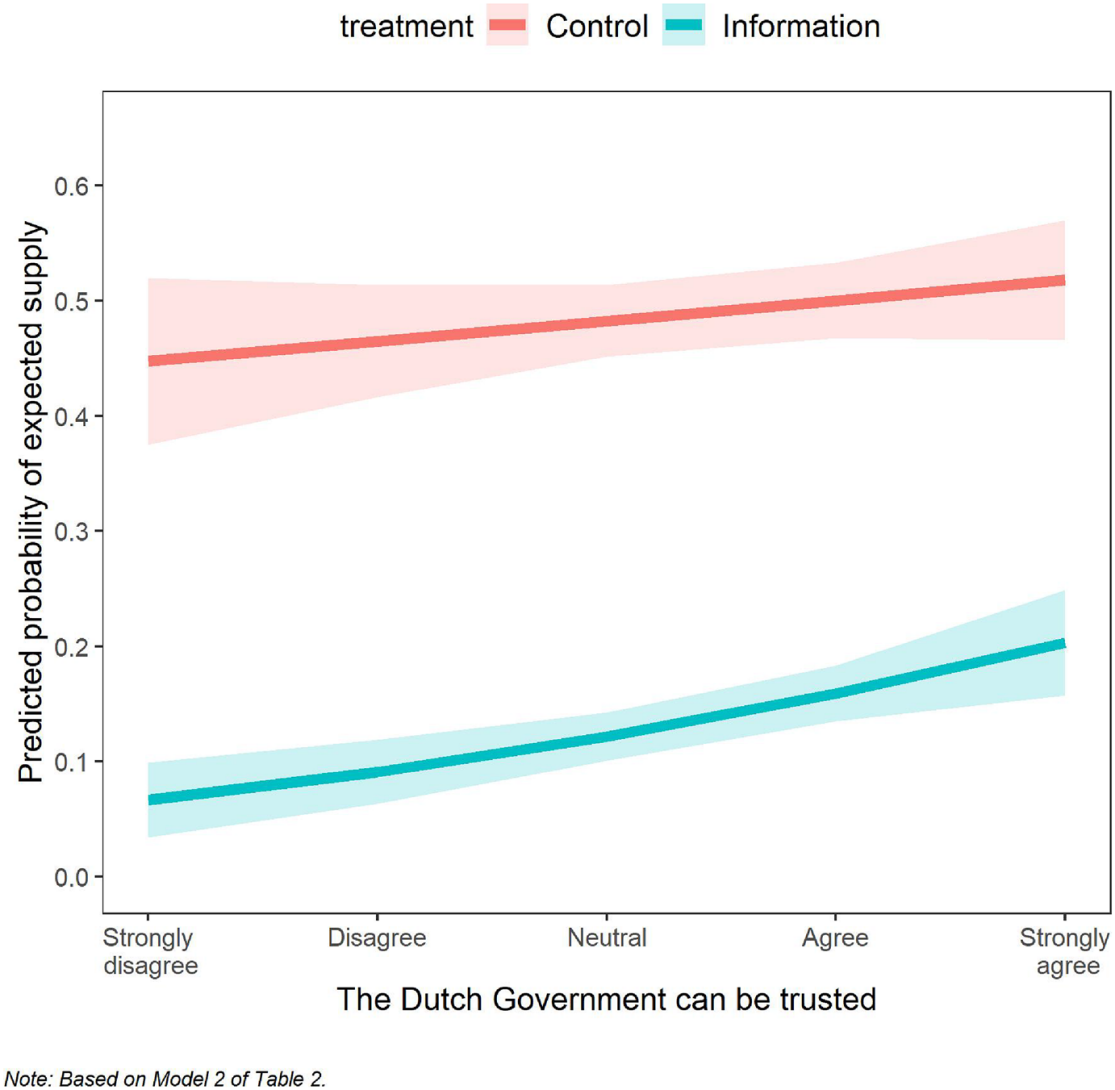
Predicted probability of expected supply by trust in government and treatment

Furthermore, we performed an ordered probit regression to examine the treatment effect of information on expected platform use under different specifications with control variables, following the preregistration. The regression analysis also allows for an inspection of the hypothesized interaction effect between the treatment and trust in institutions. Table 2 presents the results. Model 1 includes only the treatment dummy. In Model 2, we examine an interaction between the treatment dummy and trust in government. Model 3 includes the other preregistered control variables. Model 4 controls for self-reported knowledge on platform tax regulations (response to the item *How well are you aware of tax regulations regarding income earned on gig platforms?*). We find a significantly negative estimate for the Information treatment dummy across all specifications, which confirms Hypothesis 1. We find no support for Hypothesis 2: the coefficient of the interaction between the Information dummy and Trust in government is not significant in Model 2 and Model 3. Figure 2 visualizes this result.

**Table 2:** Ordered probit regressions of expected supply

	DV: expected supply			
	(1)	(2)	(3)	(4)
Information treatment (ref = Control)	− 0.537***	− 0.549***	− 0.552***	− 0.560***
	(0.094)	(0.096)	(0.097)	(0.097)
Information treatment × trust in government		0.058	0.055	0.042
		(0.087)	(0.089)	(0.090)
Trust in government		0.038	0.023	0.017
		(0.072)	(0.074)	(0.075)
Trust in platforms			0.004	− 0.009
			(0.062)	(0.062)
Gender (1 = female)			− 0.105	− 0.084
			(0.101)	(0.102)
Age			− 0.013	− 0.014
			(0.008)	(0.008)
Full-time platform work			0.101	0.111
			(0.109)	(0.109)
Hours/week working on platform			− 0.004	− 0.004
			(0.006)	(0.006)
Self-reported tax knowledge				0.091
				(0.060)
Log likelihood	− 618.2	− 594.9	− 590.1	− 588.8
Pseudo R(McFadden)	0.028	0.031	0.037	0.039
Observations	626	603	601	601

Robust standard errors are given in parentheses *p < 0.05; **p < 0.01, ***p < 0.001. Model 2 (3) excludes 23 (2) subjects who answered Rather not say to the trust in government (platforms) question. For robustness, we ran Models 1 and 2 without these subjects; the results do not change (see Table C1). Trust in government and trust in platforms are mean-centered. Order of expected supply: less < same < more

As a robustness check, we conducted a similar analysis (ordered probit regression with interaction of treatment × trust, see Table C2) with the two alternative trust variables (Trust in platforms and General trust) where we find no significant interaction effects either. Note that the trust questions were asked before the treatment information; hence, information cannot bias reported trust levels.

As a next exploratory step in our analyses, we examine the characteristics of the respondents who expect to work *more* via platforms in the coming 2 years, since the proportion of respondents who expect to work *less* is approximately equal across treatments. What characterizes the respondents who expect to work *more*—in other words, optimistic respondents? And is the treatment effect moderated by such individual characteristics?

**Fig. 3:**
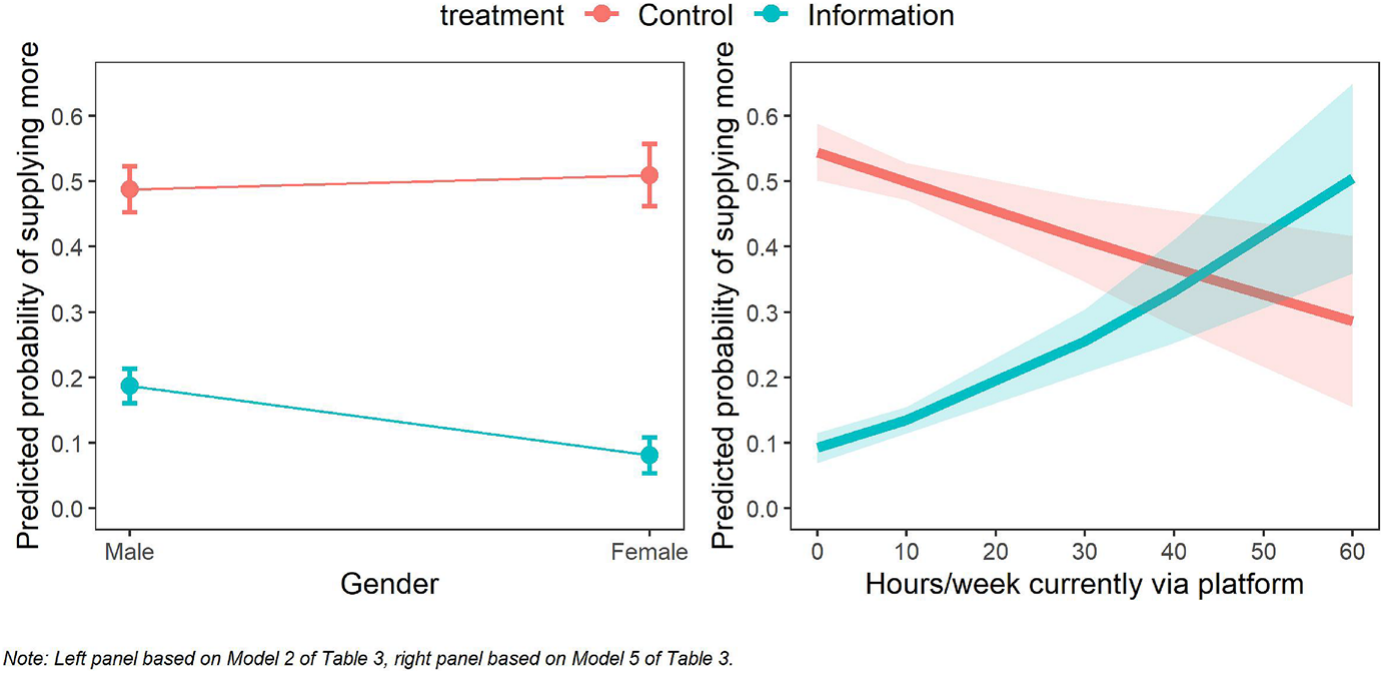
Predicted probabilities of supplying more on platforms in the coming 2 years, by treatment

To examine this question, we estimated probit regressions where the dependent variable is a dummy indicating expected supply = *more*, and the reference category is expected supply *equal* or *less*. Table 3 shows the results. Across all models, the coefficient of the Information treatment dummy is negative, which is in line with the results in Table 2. That is, the probability that respondents indicate they expect to work *more* (as compared to *equal* or *less*) is generally lower in the Information treatment compared to the Control treatment. We find a significant interaction effect between treatment and gender in Model 2. This suggests that the negative effect of information on the expected supply dummy (focusing on the contrast between supply of *more* labor versus supply of *equal* or *less* labor) is larger for women than for men. The left panel of Fig. 3 visualizes this. However, the interaction effect between the Information treatment and gender does not appear in Model 7 (which includes all interaction effects) nor in the robustness check where we used a linear (OLS) specification (Table C4). The results should, therefore, be interpreted with caution.

**Table 3:** Probit regressions of expect to work more

	DV: expected supply (1 = more, ref = equal or less)						
	(1)	(2)	(3)	(4)	(5)	(6)	(7)
Constant	− 0.012	− 0.031	0.460*	− 0.017	0.111	− 0.223	0.233
	(0.071)	(0.088)	(0.269)	(0.085)	(0.109)	(0.317)	(0.440)
Information treatment (ref = Control)	− 1.010***	− 0.858***	− 1.566***	− 1.120***	− 1.436***	− 1.141*	− 1.697**
	(0.112)	(0.133)	(0.396)	(0.136)	(0.182)	(0.530)	(0.649)
Gender (1 = female)		0.054					0.073
		(0.149)					(0.154)
Information × Female		− 0.564*					− 0.515
		(0.256)					(0.266)
Age			− 0.022				− 0.020
			(0.012)				(0.012)
Information × age			0.026				0.021
			(0.018)				(0.018)
Full-time platform work				0.017			0.043
				(0.155)			(0.162)
Information × Full-time platform work				0.351			0.213
				(0.241)			(0.257)
Hours/week working on platform					− 0.011		− 0.009
					(0.007)		(0.008)
Information × hours/w working on platform					0.034**		0.028*
					(0.011)		(0.012)
Self-reported tax knowledge						0.058	0.069
						(0.086)	(0.089)
Information × self-reported tax knowledge						0.033	− 0.012
						(0.140)	(0.137)
Log likelihood	− 351.1	− 347.8	− 349.1	− 349.1	− 345.8	− 350.4	− 340.3
Pseudo R(McFadden)	0.11	0.118	0.115	0.115	0.123	0.112	0.137
Observations	626	626	626	626	626	626	626

Robust standard errors are given in parentheses *p < 0.05 **p < 0.01; ***p< 0.001

The significantly positive coefficient of the interaction in Model 5 indicates that the negative effect of information on expected supply (again focusing on the contrast between supply of *more* labor versus supply of *equal* or *less* labor) decreases by the number of hours a respondent is currently working via platforms. In other words, those who currently work few hours via platforms are more affected by the information about regulation change than those who currently work many hours via platforms. This finding is visualized in the right panel of Fig. 3 and may be related to the fact that individuals who work many hours on platforms have little opportunity for changes in the future. Note that we did not restrict the number of hours in a workweek to 40, which is the most common for full-time employment in the Netherlands, because the flexibility of gig economy work allows for work outside office hours. Indeed, some of the responses to this survey question are well above the 40 h (minimum = 0, maximum = 56). Those who currently work few hours via platforms, on the other hand, may be generally optimistic about working more hours via platforms in the future, but when they are informed about changing regulations*,* this optimism fades.

In Model 6, we examine the interaction between self-reported knowledge on platform taxation and the information treatment. We find no significant interaction coefficient, which suggests that the information treatment has the same effect on all participants (regardless of whether they believe themselves to be well-informed).

## Concluding discussion

This research used a survey among a large group of platform workers (*N* = 626) in the Netherlands to examine how they view a proposed regulation change in the taxation system in the context of the platform economy. Our analysis considered the proportion of platform income out of the workers’ total impact and several measures of trust (generalized, in the government, and in platforms). The survey included an experimental manipulation of information, either providing participants with information about the upcoming taxation change or not. The results show that informing respondents about the regulation negatively affects expected supply of labor on the platform. We found no interaction between the information treatment and the measures of trust on expected future supply. This result appears consistent with other research in several European countries, which found no interactions between changes in monitoring power and trust in institutions (Kogler et al., 2013; Wahl et al., 2010). Furthermore, we found two distinct groups of respondents, those who were mainly positive about the upcoming regulation change (52%, in line with promotion focus) and those who selected only negative answers (41%, in line with prevention focus). Recent work shows that messages matching a recipient’s regulatory focus are more likely to be effective (Holler et al., 2008; Roczniewska & Higgins, 2019) and that this regulatory focus can interact with generalized trust (Keller et al., 2015). It could be interesting to assess regulatory focus with the regulatory focus scale (Fellner et al., 2007) in future research to examine this interaction more closely, particularly because regulatory focus seems to be context dependent, which challenges the idea that losses fundamentally loom larger than gains (Gal & Rucker, 2018).

Currently, many people may not know that they need to pay income tax on their platform revenues. It could also be the case that people are aware of income tax in relation to platform revenues, but that they are not aware of the details, such as the threshold level. Information about tax awareness regarding the platform economy is currently lacking, both regarding taxpayers (providers) as well as users. This study is one of the first empirical studies among the relevant decision-makers: gig economy workers in the Netherlands, and the first to look at the expected effect of a proposed regulatory change at the EU level. We find that informing gig workers about the upcoming regulatory change decreases expected supply of labor, irrespective of respondents’ prior knowledge of tax regulations regarding income on gig platforms.

One limitation of this study is that the main dependent variable is self-reported expected future behavior. This may give a good indication regarding optimism, but it may not be the best indicator for actual behavior in the future. We recommend that future research examines the effect of regulatory interventions on actual platform economy supply by closely collaborating with platforms in a field experiment. However, we believe that the current study can be informative for policymakers and can serve as a prerequisite for such a future field experiment. Another limitation is the possibility of a demand effect, even though we tried to minimize this by asking opinions about the regulation referring to the majority of platform workers. Furthermore, we asked about expected future labor supply, rather than future tax compliance, to encourage honest responses. The assumption is that the regulation change may halt increasing working hours at the platform, which allows for more working hours in other, unregulated labor markets. This does not translate one-to-one into tax compliance, but note that tax evasion research is a sensitive topic, which calls for a balance of demand effects, attrition, and dishonest answers. Finally, future research can attempt to replicate the finding that information about a regulation change has a negative effect on expected future labor supply and shed light on the mechanisms underlying this diminished optimism. Given the time constraints for the current voluntary survey, we took care to limit the number of questions. However, future surveys could explicitly measure intentions to work more hours in other and unregulated markets, as well as the type of platform jobs.

This study suggests the following main takeaways for policymakers. It is crucial to communicate the proposed regulatory changes clearly to platform providers. However, there is no need to specifically target citizens with low trust in the government when developing communication about regulation changes, at least in the Dutch context. This finding is in line with other recent literature on health compliance behavior, where trusting the government generated only modest effects (Bicchieri et al., 2021).

Our results seem to suggest that a certain subgroup of informed platform providers respond with diminished optimism, namely women and part-time workers. Previous survey research has shown that there are several distinct subgroups of gig economy providers and that they do different types of jobs (for example students doing IT or design consultancy and single mothers combining different cleaning positions) (Huws et al., 2017). However, the current study did not survey which types of jobs were performed. Future research could create different profiles of gig economy providers and test their responses to the proposed regulatory change, which is useful input to design clear communication about rights and obligations. In addition, the identification of job types could allow for a clear interpretation of the different responses across subgroups. Finally, future work could assess the effect of monitoring power on platform labor supply by an information treatment which is more explicit about a shift in the power of authorities, and the inclusion of a manipulation check. Given our findings, we expect that a stronger manipulation of monitoring power could have even more pronounced effects on platform users’ intended labor supply.

## Supplementary Information

Below is the link to the electronic supplementary material.

Supplementary file1 (DOCX 267 KB)

## Data Availability

The replication and supplementary material for the study is available at 10.17605/OSF.IO/6W87B.

## Footnotes

1 Note that taxpayers (platform users) will therefore experience this change from 2024 onwards. See https://download.belastingdienst.nl/belastingdienst/docs/dac7-tijdlijn-wetgeving-al4841z1ed.pdf..

2 Hypothesis 1 was somewhat differently phrased in our preregistration, although the direction of the expected relationship was the same. Following advice from an anonymous reviewer, we revised the phrasing of H1 to explicitly mention details about the regulation change in question.

3 Note that we preregistered *trust in institutions* which refers to the same survey item “Do you agree/disagree to the statement: The Dutch government can be trusted”.
