# Supplementary material for: Information about changes in platform economy taxation diminishes optimism regarding future use
Source: J Econ Sci Assoc. 2024 Mar 14;10(2):330–45. doi: 10.1007/s40881-024-00160-y (PMC11967534; doi:10.1007/s40881-024-00160-y)
Supplement: Supplementary file 1 — Supplementary file1 (DOCX 267 KB) [file 40881_2024_160_MOESM1_ESM.docx]

## Appendix A: Invitation email text (translated from Dutch)


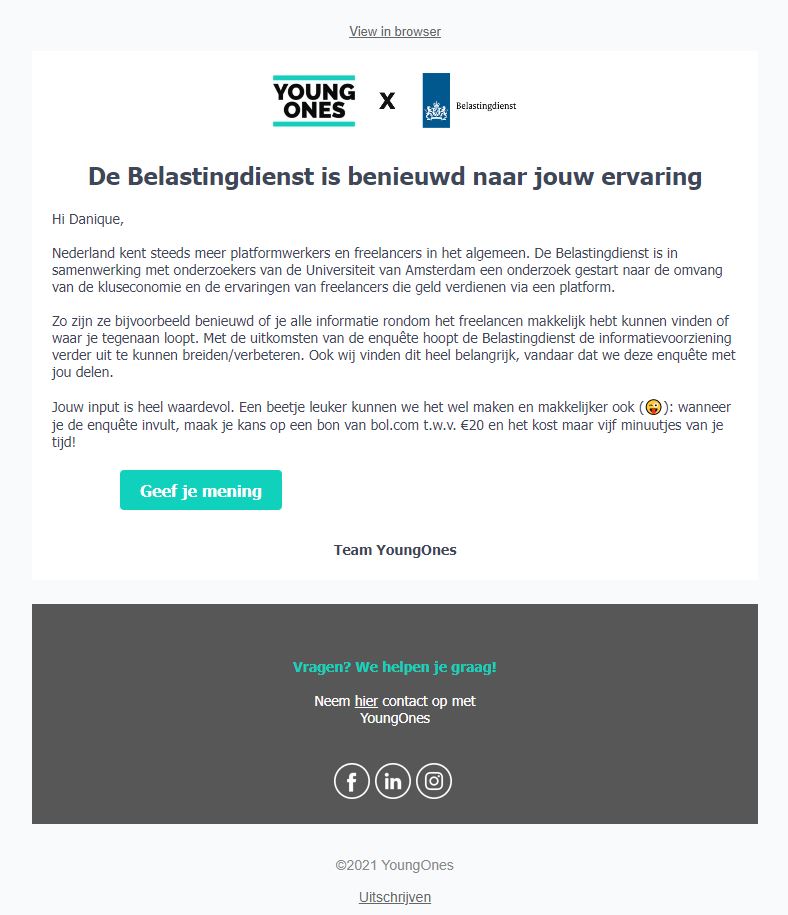


Figure A 1: Screenshot of invitation email

**The Dutch Tax Authority is curious about your experience**

Hi [name],

More and more people in the Netherlands perform gigs and become self-employed. The Dutch Tax Authority partners with researchers of the University of Amsterdam to examine the size of the gig economy and the experiences of self-employed gig workers.

For example, they would like to know how easy it was for you to find all information about freelance jobs or what information is missing. The results of the survey will be used to improve the website and service of the Dutch Tax Authority. We also find this important, which is why we would like to share this survey with you.

Your input is very valuable. We can make it a little more easy and fun: if you complete the survey you are eligible for a 20 euro Bol.com voucher. It will only take five minutes of your time!

Give your opinion!

## Appendix B: All survey items (translated from Dutch)

*Note that only questions indicated with an asterisk (*) were analyzed in the current study. The other questions were analyzed in a policy report for the YoungOnes platform and the Dutch Tax Authority (to help improve their website and service). This list of survey items reflects the order in which the questions were asked.*

**[consent]*

Welcome. Thank you for participating in this research about the gig economy by YoungOnes and the University of Amsterdam. The goal of this research is to understand the size of the gig economy and its workers.

This survey is administered via Qualtrics, and the data will be stored on an European server. Only researchers of the University of Amsterdam will have access to the data. A summary of the results will be shared with YoungOnes (in other words, not your individual answers).

This procedure is in line with the European regulations (GDPR) and the ethical committee of the Faculty of Economics and Business at the University of Amsterdam. **Your results will be treated anonymously.** This means that your answers cannot be traced to you individually.

Your participation is voluntary and you can quit any moment by closing your browser. If you proceed to the next page, you consent to participation.

*[platform]*

What is the main reason to work via YoungOnes?

- Making extra money
- Meeting new people
- If you are self-employed: finding new/extra customers
- Increasing job opportunities
- Other, namely: [open answer box]

**[hours_platform]*

How many hours per week do you work approximately via YoungOnes? We are interested in the number of hours you get paid, not the number of hours you are available.

[open answer box, accepting numbers between 0 - 168]

**[hours_platform_prop]*

How does your YoungOnes income relate to your total income?

- I do not earn anything (yet) (costs are higher than benefits)
- Less than half of my total income
- Approximately half of my total income
- More than half of my total income
- It is my only income

*[satisfaction]*

How satisfied are you with YoungOnes work?

- Extremely unsatisfied
- Unsatisfied
- Not satisfied/ not unsatisfied
- Satisfied
- Extremely satisfied

*[satisfaction_other]*

Can you explain why you are *[satisfaction]* about YoungOnes work?

[open answer box]

*[others]*

Are you signed up at other gig economy platforms to find gigs?

- Yes
- No

*[num_others, only if ‘Yes’ is answered at ‘others’]*

At how many other platforms are you offering your services?

[open answer box, accepting numeric answers]

*[hours_others]*

Approximately how many hours per week do you work via other platforms? (In total, so for all other platforms combined)

[open answer box, accepting numbers between 0 - 168]

**[trust]*

Please let us know how much you agree with the following statements:

*[trust_general]* Most other people can be trusted.

*[trust_government]* The Dutch government can be trusted.

*[trust_platforms]* Digital platforms can be trusted

- Totally agree
- Agree
- Do not agree/do not disagree
- Disagree
- Totally disagree
- Rather not say

**[self_report_know]*

How well are you aware of tax regulations with regard to income earned on gig platforms?

- Very uninformed
- Uninformed
- Not informed/not uninformed
- Informed
- Very informed

*[info_search, only displayed if ‘*Very uninformed*’, ‘*Uninformed*’ or ‘*Not informed/not uninformed*’ is selected]*

You indicated that you are [self_report_know] about tax regulations with regard to income earned on gig platforms. Below we list several ways to find more information. Where would you start searching for information? Multiple answers are allowed.

- Website of YoungOnes
- Website of the Dutch Tax Authority
- Website of the Dutch Chamber of Commerce
- Social media
- Family or friends
- Other, namely: [open box for answers]

*[info_searched, only displayed if ‘*Informed*’ or ‘*Very informed*’ is selected]*

You indicated that you are [self_report_know] about tax regulations with regard to income earned on gig platforms. Below we list several ways to find more information. Where did you find this information? Multiple answers are allowed.

- Website of YoungOnes
- Website of the Dutch Tax Authority
- Website of the Dutch Chamber of Commerce
- Social media
- Family or friends
- Other, namely: [open box for answers]

*[experience_BD_site, only displayed if ‘*Website of the Dutch Tax Authority’ *is selected]*

We would like to know how you experienced the website of the Dutch Tax Authority when searching for information about gig platform income. Please indicate whether you agree or disagree with the following statements:

*[info_BD_fast]* The information I was looking for could be found (quickly).

*[info_BD_understand]* The information I was looking for was easy to understand.

*[info_BD_extensive]* The information I was looking for was extensive.

*[info_BD_jargon]* The information I was looking for contained a lot of jargon.

- Totally agree
- Agree
- Do not agree/do not disagree
- Disagree
- Totally disagree
- Rather not say

*[pref_info]*

There are several ways through which you can find information about gig platform work and the accompanying tax regulations. Which way do you prefer?

- Looking it up on my own account
- Being informed by the platforms as soon as I register
- Being informed by the platforms as soon as I completed my first gig
- Being informed by the Dutch Tax Authority
- Other, namely: [open box for answers]

*[pref_info_channel]*

Which channel do you prefer for information about gig platform work and the accompanying tax regulations?

- E-mail
- Social media
- Text message or Whatsapp message on my phone
- Regular mail
- Other, namely: [open box for answers]

*[pref_info_moment]*

What point in time is the most likely one to look for information about gig platform work and the accompanying tax regulations?

- Before registering at one or multiple platforms
- After registering at one or multiple platforms, but before starting a gig
- After I completed my first gig at a platform
- At the time I have to fill my tax returns
- Other, namely: [open box for answers]

*[info_platform_clear]*

Did you have complete information before you started your first gig at YoungOnes, or was any information missing, for example about tax regulations?

- All was clear
- Not everything was clear

*[info_platform_unclear, only displayed if* ‘Not everything was clear’ *was selected]*

You indicate that not everything was clear. Which information were you missing at the time? Please giver you answers in some short sentences or key words. (The answers to this question will be used to improve the information on the YoungOnes website).

[open box for answers]

*[information treatment]*

In March 2021, the EU passed a law that requires digital platforms to report the income of their platform workers to the respective national tax authorities, starting January 1st, 2023. This information may be used to prepopulate tax forms (starting 2024) similar to the tax forms of citizens in wage labor.

*[*regulation_opinion]*

What do you think most gig workers think of this? (multiple answers possible)

- Not necessary, I can handle this myself
- Annoying, now I need to pay more taxes
- Great, this saves quite some administrative work
- Fair, this way all gig workers are taxed equally
- Other, namely: [open box for answers]

**[supply_intervention]*

Do you think the new EU law will change something to the number of hours you will work via a platform in the coming two years?

- Yes, I think I will work more via platforms
- Yes, I think I will work less via platforms
- No, I expect no change

**[supply_control]*

Do you think anything will change in the number of hours you will work via platforms in the coming two years?

- Yes, I think I will work more via platforms
- Yes, I think I will work less via platforms
- No, I expect no change

Finally, some general questions.

**[gender]*

What is your gender?

- Female
- Male
- Other
- Rather not say

**[age]*

What is your age?

[dropdown menu: 18 to 120 years] – because 16 and 17 were missing (but possible at YoungOnes) and some participants noted their age in the general comments box at the end, we recoded 116 to 16 and 117 to 17, and hand-coded those who commented being 16 of 17 years old.

**[occupation]*

Could you indicate which of the following situations describes you the best?

- I have a full time job on payroll (36 hours or more per week)
- I have a part time job or multiple part time jobs on payroll
- I work via one or multiple gig platforms
- I am self-employed
- I am a student
- I have a disability that stops me from joining the workforce
- I am currently looking for work (and not a student)
- I do full time voluntary work
- I am retired
- Other, namely: [open box for answers]

**[income]*

What is (approximately) your after tax income of your household?

- Less than €999
- Between €1000 and €1999
- Between €2000 and €2999
- Between €3000 and €3999
- Between €4000 and €4999
- €5000 or more
- Rather not say

*[household]*

How many adults are in your household?

- 1
- 2
- 3
- 4
- 5
- Other, namely: [text box for answers]

*[edu]*

What is the highest degree you have completed?

- No diploma / Primary school / Secondary school
- Vocational ecucation / MBO
- HBO / University Bachelor
- University Master or more (PhD / PdEng)
- Other, namely: [open box for answers]

# Appendix C: Robustness checks

*Table C1: Table 2 excluding incomplete responses.*

|  | DV: expected supply | | | |
| --- | --- | --- | --- | --- |
|  | (1) | (2) | (3) | (4) |
| Information treatment (ref = Control) | -0.551*** | -0.552*** | -0.552*** | -0.560*** |
|  | (0.096) | (0.096) | (0.097) | (0.097) |
| Information treatment × Trust in government |  | 0.057 | 0.055 | 0.042 |
|  |  | (0.088) | (0.089) | (0.090) |
| Trust in government |  | 0.041 | 0.023 | 0.017 |
|  |  | (0.073) | (0.074) | (0.075) |
| Trust in platforms |  |  | 0.004 | -0.009 |
|  |  |  | (0.062) | (0.062) |
| Hours/week working on platform |  |  | -0.004 | -0.004 |
|  |  |  | (0.006) | (0.006) |
| Gender (1 = female) |  |  | -0.105 | -0.084 |
|  |  |  | (0.101) | (0.102) |
| Age |  |  | -0.013 | -0.014 |
|  |  |  | (0.008) | (0.008) |
| Full-time platform work |  |  | 0.101 | 0.111 |
|  |  |  | (0.109) | (0.109) |
| Self-reported tax knowledge |  |  |  | 0.091 |
|  |  |  |  | (0.060) |
| Log likelihood | -594.9 | -593.5 | -590.1 | -588.8 |
| Pseudo R(McFadden) | 0.029 | 0.031 | 0.037 | 0.039 |
| Observations | 601 | 601 | 601 | 601 |
| *Notes*: Robust standard errors are given in parentheses *p<0.05 **p<0.01; ***p<0.001. | | | | |

*Table C2: Table 2 with alternative trust variables.*

|  | | | |
| --- | --- | --- | --- |
|  | DV: expected supply | | |
|  |  | | |
|  |  | | |
|  | (1) | (2) | (3) |
|  | | | |
| Information treatment (ref = Control) | -0.549*** | -0.549*** | -0.536*** |
|  | (0.096) | (0.095) | (0.095) |
| Information treatment × Trust in government | 0.058 |  |  |
|  | (0.087) |  |  |
| Information treatment × Trust in platforms |  | -0.086 |  |
|  |  | (0.116) |  |
| Information treatment × General trust |  |  | -0.080 |
|  |  |  | (0.104) |
| Trust in government | 0.038 |  |  |
|  | (0.072) |  |  |
| Trust in platforms |  | 0.065 |  |
|  |  | (0.099) |  |
| General trust |  |  | 0.036 |
|  |  |  | (0.084) |
|  | | | |
| Log likelihood | -594.9 | -605.9 | -601.6 |
| Pseudo R(McFadden) | 0.031 | 0.029 | 0.028 |
| Observations | 603 | 613 | 610 |
|  | | | |
| *Notes*: Robust standard errors are given in parentheses *p<0.05 **p<0.01; ***p<0.001. | | | |

*Table C3: Table 2 with alternative full time dummy.*

|  | DV: expected supply | | | |
| --- | --- | --- | --- | --- |
|  | (1) | (2) | (3) | (4) |
| Information treatment (ref = Control) | -0.537*** | -0.549*** | -0.564*** | -0.572*** |
|  | (0.094) | (0.096) | (0.096) | (0.097) |
| Information treatment × Trust in government |  | 0.087 | 0.090 | 0.090 |
|  |  | (0.038) | (0.016) | (0.010) |
| Trust in government |  | 0.038 | 0.016 | 0.010 |
|  |  | (0.072) | (0.075) | (0.076) |
| Trust in platforms |  |  | 0.005 | -0.007 |
|  |  |  | (0.062) | (0.063) |
| Hours/week working on platform |  |  | -0.004 | -0.003 |
|  |  |  | (0.007) | (0.007) |
| Gender (1 = female) |  |  | -0.094 | -0.074 |
|  |  |  | (0.101) | (0.102) |
| Age |  |  | -0.014 | -0.014 |
|  |  |  | (0.008) | (0.008) |
| Full-time platform work |  |  | -0.489 | -0.480 |
|  |  |  | (0.348) | (0.351) |
| Self-reported tax knowledge |  |  |  | 0.086 |
|  |  |  |  | (0.061) |
| Log likelihood | -618.2 | -594.9 | -589.3 | -588.1 |
| Pseudo R(McFadden) | 0.028 | 0.031 | 0.038 | 0.040 |
| Observations | 626 | 603 | 601 | 601 |
| *Notes*: Robust standard errors are given in parentheses *p<0.05 **p<0.01; ***p<0.001. The original dummy for full-time workers was constructed on the answers to the question “How does your platform income related to your total income” – participants who answered “It is my only income” were coded as 1, all others as 0. However, when we tabulate this full-time platform income dummy with the number of hours on the platform, we find that these participants are not necessarily working 40 hours or more. Therefore, we constructed another dummy for participants who work 40 hours or more. This table shows Table 2 with the alternative full time dummy. | | | | |

*Table C4: Linear regressions (OLS) of expected supply (robustness check Table 2).*

|  | DV: expected supply | | | |
| --- | --- | --- | --- | --- |
|  | (1) | (2) | (3) | (4) |
| Constant | 0.310*** | 0.223 | 0.439* | 0.286 |
|  | (0.038) | (0.133) | (0.191) | (0.213) |
| Information treatment (ref = Control) | -0.319*** | -0.458* | -0.450* | -0.426* |
|  | (0.054) | (0.187) | (0.189) | (0.189) |
| Information treatment × Trust in government |  | 0.038 | 0.036 | 0.028 |
|  |  | (0.053) | (0.053) | (0.053) |
| Trust in government |  | 0.022 | 0.013 | 0.010 |
|  |  | (0.037) | (0.039) | (0.039) |
| Trust in platforms |  |  | 0.002 | -0.006 |
|  |  |  | (0.037) | (0.038) |
| Gender (1 = female) |  |  | -0.064 | -0.051 |
|  |  |  | (0.059) | (0.060) |
| Age |  |  | -0.008 | -0.008 |
|  |  |  | (0.004) | (0.004) |
| Full-time platform work |  |  | 0.062 | 0.068 |
|  |  |  | (0.063) | (0.063) |
| Hours/week working on platform |  |  | -0.002 | -0.002 |
|  |  |  | (0.003) | (0.003) |
| Self-reported tax knowledge |  |  |  | 0.055 |
|  |  |  |  | (0.034) |
| Observations | 626 | 603 | 601 | 601 |
| R^2^ | 0.054 | 0.061 | 0.072 | 0.076 |
| *Notes*: Robust standard errors are given in parentheses *p<0.05 **p<0.01; ***p<0.001. | | | | |

| *Table C5: Table 3 with alternative full time dummy.* | | | | | | | | |
| --- | --- | --- | --- | --- | --- | --- | --- | --- |
|  | | DV: expected supply (1 = more, ref = equal or less) | | | | | | |
|  | | (1) | (2) | (3) | (4) | (5) | (6) | (7) |
| Constant | | -0.012 | -0.031 | 0.460* | 0.021 | 0.111 | -0.223 | 0.143 |
|  | | (0.071) | (0.088) | (0.269) | (0.072) | (0.109) | (0.317) | (0.450) |
| Information treatment (ref = Control) | | -1.010*** | -0.858*** | -1.566*** | -1.072*** | -1.436*** | -1.141* | -1.551* |
|  | | (0.112) | (0.133) | (0.396) | (0.115) | (0.182) | (0.530) | (0.647) |
| Gender (1 = female) | |  | 0.054 |  |  |  |  | 0.087 |
|  | |  | (0.149) |  |  |  |  | (0.154) |
| Information × Female | |  | -0.564* |  |  |  |  | -0.522 |
|  | |  | (0.256) |  |  |  |  | (0.269) |
| Age | |  |  | -0.022 |  |  |  | -0.019 |
|  | |  |  | (0.012) |  |  |  | (0.012) |
| Information × Age | |  |  | 0.026 |  |  |  | 0.017 |
|  | |  |  | (0.018) |  |  |  | (0.018) |
| Full-time platform work | |  |  |  | -0.988* |  |  | -0.928 |
|  | |  |  |  | (0.438) |  |  | (0.559) |
| Information × Full-time platform work | |  |  |  | 1.537** |  |  | 0.731 |
|  | |  |  |  | (0.577) |  |  | (0.758) |
| Hours/week working on platform | |  |  |  |  | -0.011 |  | 0.002 |
|  | |  |  |  |  | (0.007) |  | (0.010) |
| Information × Hours/w working on platform | |  |  |  |  | 0.034** |  | 0.022 |
|  | |  |  |  |  | (0.011) |  | (0.015) |
| Self-reported tax knowledge | |  |  |  |  |  | 0.058 | 0.063 |
|  | |  |  |  |  |  | (0.086) | (0.091) |
| Information × Self-reported tax knowledge | |  |  |  |  |  | 0.033 | -0.004 |
|  | |  |  |  |  |  | (0.140) | (0.140) |
| Log likelihood | | -351.1 | -347.8 | -349.1 | -347.1 | -345.8 | -350.4 | -339.5 |
| Pseudo R(McFadden) | | 0.11 | 0.118 | 0.115 | 0.120 | 0.123 | 0.112 | 0.139 |
| Observations | | 626 | 626 | 626 | 626 | 626 | 626 | 626 |
| \| *Notes*: Robust standard errors are given in parentheses *p<0.05 **p<0.01; ***p<0.001. The original dummy for full-time workers was constructed on the answers to the question “How does your platform income related to your total income” – participants who answered “It is my only income” were coded as 1, all others as 0. However, when we tabulate this full-time platform income dummy with the number of hours on the platform, we find that these participants are not necessarily working 40 hours or more. Therefore, we constructed another dummy for participants who work 40 hours or more. This table shows Table 3 with the alternative full time dummy. \| \| --- \| | | | | | | | | |
|  | | | | | | | | |
| *Table C6: Linear regressions (OLS) of the expectation to work more (robustness check Table 3)* | | | | | | | | |
|  | | DV: expect to supply more | | | | | | |
|  | | (1) | (2) | (3) | (4) | (5) | (6) | (7) |
| Constant | | 0.495*** | 0.488*** | 0.679*** | 0.493*** | 0.544*** | 0.412*** | 0.586*** |
|  | | (0.025) | (0.031) | (0.085) | (0.029) | (0.038) | (0.111) | (0.145) |
| Information treatment (ref = Control) | | -0.342*** | -0.301*** | -0.547*** | -0.365*** | -0.465*** | -0.338^*^ | -0.549** |
|  | | (0.035) | (0.043) | (0.116) | (0.041) | (0.054) | (0.153) | (0.192) |
| Gender (1 = female) | |  | 0.021 |  |  |  |  | 0.028 |
|  | |  | (0.052) |  |  |  |  | (0.053) |
| Information × Female | |  | -0.128 |  |  |  |  | -0.112 |
|  | |  | (0.074) |  |  |  |  | (0.076) |
| Age | |  |  | -0.008* |  |  |  | -0.008 |
|  | |  |  | (0.004) |  |  |  | (0.004) |
| Information × Age | |  |  | 0.009 |  |  |  | 0.008 |
|  | |  |  | (0.005) |  |  |  | (0.005) |
| Full-time platform work | |  |  |  | 0.007 |  |  | 0.016 |
|  | |  |  |  | (0.054) |  |  | (0.055) |
| Information × Full-time platform work | |  |  |  | 0.086 |  |  | 0.040 |
|  | |  |  |  | (0.077) |  |  | (0.079) |
| Hours/week working on platform | |  |  |  |  | -0.004 |  | -0.004 |
|  | |  |  |  |  | (0.003) |  | (0.003) |
| Information × Hours/w working on platform | |  |  |  |  | 0.010** |  | 0.008* |
|  | |  |  |  |  | (0.004) |  | (0.004) |
| Self-reported tax knowledge | |  |  |  |  |  | 0.023 | 0.027 |
|  | |  |  |  |  |  | (0.030) | (0.030) |
| Information × Self-reported tax knowledge | |  |  |  |  |  | -0.002 | -0.011 |
|  | |  |  |  |  |  | (0.041) | (0.041) |
| Observations | | 626 | 626 | 626 | 626 | 626 | 626 | 626 |
| R^2^ | | 0.133 | 0.139 | 0.140 | 0.137 | 0.146 | 0.135 | 0.159 |
| \| *Notes*: Robust standard errors are given in parentheses *p<0.05 **p<0.01; ***p<0.001. \| \| --- \| | | | | | | | | |
|  | | | | | | | | |

**Appendix D: Information for sellers on Tax Authority Website (translated from Dutch)**

#
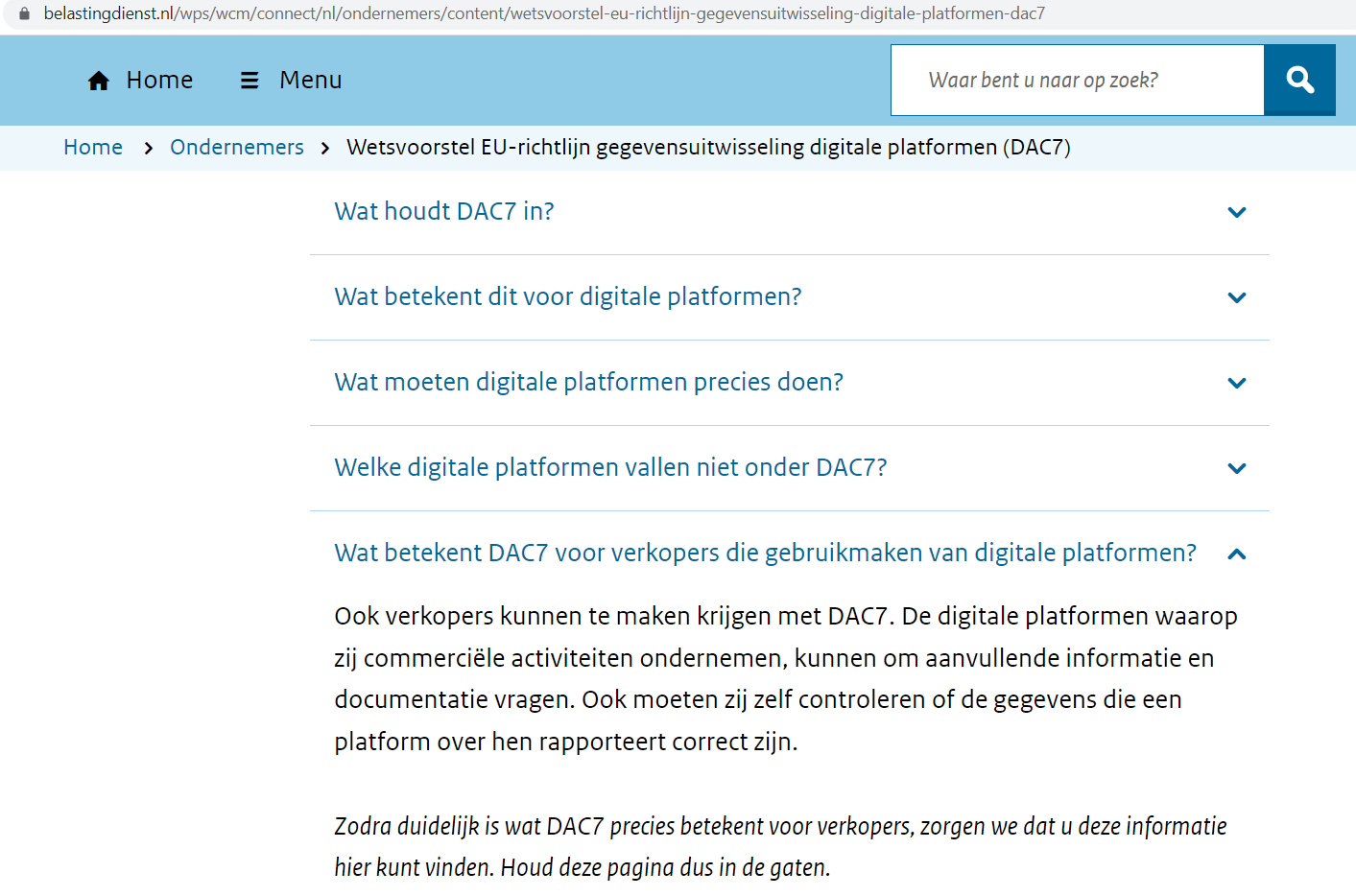


Figure D 1: Information for sellers on Tax Authority Website (retrieved in December 2022)

**What does DAC7 mean for sellers who use digital platforms?**

Sellers may have to deal with DAC7 as well. The digital platforms used for commercial activities may ask for additional information and documentation. Furthermore, sellers need to check whether the information reported by the platform is correct.

*As soon as it is clear what DAC7 means exactly to sellers, we will make sure that you will find this information here. Please keep track of this page.*
